# Year in review 2013: *Critical Care* - nephrology

**DOI:** 10.1186/s13054-014-0574-1

**Published:** 2014-10-21

**Authors:** Zaccaria Ricci, Matteo Di Nardo, Claudio Ronco

**Affiliations:** Department of Pediatric Cardiac Surgery, Bambino Gesù Children’s Hospital, IRCCS, 00165 Rome, Italy; Department of Emergency, Pediatric Intensive Care Unit, Bambino Gesù Children’s Hospital, IRCCS, 00165 Rome, Italy; Department of Nephrology, Dialysis and Transplantation, S. Bortolo Hospital, 36100 Vicenza, Italy; International Renal Research Institute, 36100, Vicenza, Italy

## Abstract

We review original research in the field of critical care nephrology accepted or published in 2013 in *Critical Care* and, when considered relevant or linked to these studies, in other journals. Three main topics have been identified and are discussed for a rapid overview: acute kidney injury (diagnosis, risk factors and outcome); timing and modality of renal replacement therapy; and extracorporeal membrane oxygenation and renal dysfunction.

## Introduction

Acute kidney injury (AKI) is a common and severe complication of critical illness associated with death and disability. The symptoms and clinical consequences of AKI can be quite similar regardless of whether the etiology is predominantly within the kidney or predominantly outside the kidney; the AKI syndrome encompasses both structural injury as well as acute functional impairment. The costs of care for these patients are very high and research on AKI is thus focusing on prevention, early detection and treatment. The non-profit foundation Kidney Disease: Improving Global Outcome (KDIGO), managed by the National Kidney Foundation with the aim of developing and implementing guidelines, completed in 2012 the first international, multidisciplinary, clinical practice guidelines for AKI [1,2]. This workgroup utilized previously existing criteria to try and unify and develop all aspects, both clarified and uncertain, of AKI and critical care nephrology. The Acute Dialysis Quality Initiative group had designed the RIFLE (Risk, Injury, Failure, Loss of function and End-stage kidney disease) system for diagnosis and classification through a broad consensus of experts back in 2004 [3]. This classification was also modified for pediatric patients in order to better classify small children with AKI (pediatric RIFLE) [4]. More recently, the Acute Kidney Injury Network (AKIN) endorsed the RIFLE criteria with a modification to include changes in serum creatinine (sCr; ≥0.3 mg/dl or 26.5 μmol/l) when they occur within a 48-hour period [5]. Unfortunately, the existing criteria, while useful and widely validated, are still limited by relatively scarce and non-uniform clinical application [6]. The KDIGO workgroup focused their effort into reconciling all the different criteria and providing a comprehensive set of guidelines and new definitive AKI criteria (Table 1).

**Table 1:** **Kidney disease: improving global outcome (KDIGO) classification for acute kidney injury diagnosis**

	**Stage 1**	**Stage 2**	**Stage 3**
KDIGO (2012)	↑sCr ≥0.3 mg/dl (26.5 μmol/l) within 48 hours	↑sCr >2.0-2.9 × baseline	sCr ≥ 4.0 mg/dl (353.6 μmol/L)
	OR	OR	OR
	1.5-1.9 × baseline within a week	UO <0.5 ml/kg/hour for ≥12 hours	↑sCr >3.0 × baseline
	OR		OR
	UO <0.5 ml/kg/hour for 6–12 hours		UO <0.3 ml/kg/hour for 24 hours
			OR
			Anuria for 12 hours
			OR
			Initiation RRT
			OR
			In patients <18 years old, ↓ in eGFR to <35 ml/minute/1.73 m^2^

Up arrows indicate increase; down arrows indicate decrease. eGFR, estimated glomerular filtration rate; RRT, renal replacement therapy; sCr, serum creatinine; UO, urine output.

## Diagnosis of acute kidney injury

Apart from the specific issue of AKI classification, accurate assessment of kidney function in the critically ill patient plays an important role in diagnosing AKI as well as ensuring the appropriate prescription and dosing of drugs and the timely application of therapeutic strategies. sCr is used daily as a marker for kidney function [7], although it may not be suitable for this purpose as it is affected by factors other than kidney function. It is well known that creatinine is metabolized from creatine, which is released by the muscles; therefore, muscle mass and metabolic transformation of creatine have an impact on sCr concentration. In addition, age, gender and race all affect muscle mass and, in turn, sCr concentrations. In critically ill patients, creatinine production may be decreased because of immobilization and malnutrition or increased because of catabolic illness. Increases in total body water, common in these patients, increases the distribution volume of creatinine and attenuates the increase in sCr caused by AKI. Bragadottir and colleagues [8] also showed that daily changes in serum creatinine as a surrogate of glomerular filtration rate poorly reflect changes in kidney function in patients with AKI. Other groups have investigated the use of urinary indices, such as the fractional excretion of sodium and the fractional excretion of urea (FeUrea), for the differential diagnosis of different forms of AKI [9-12]. Pons and coworkers [9] performed a prospective multicenter observational study enrolling 244 consecutive patients, including 97 without AKI, 54 with transient AKI and 93 with persistent AKI. Urinary sodium, urea and creatinine were measured at ICU admission (H0) and every 6 hours during the first 24 hours in the ICU (H6, H12, H18 and H24). Transient AKI was defined as AKI with a cause for renal hypoperfusion and reversal within 3 days. Even though significant increases from H0 to H24 were noted in FeUrea (median 31% (22 to 41%) at H0; 39% (29 to 48%) at H24; *P* <0.0001), urinary urea/plasma urea ratio (median 15 (7 to 28) at H0; 20 (9 to 40) at H24; *P* <0.0001), and urinary creatinine/plasma creatinine ratio (median 50 (24 to 101) at H0; 57 (29 to 104) at H24; *P* = 0.01), fractional excretion of sodium did not change significantly during the first 24 hours in the ICU (*P* = 0.13). Neither urinary index values at ICU admission nor changes in urinary indices between H0 and H24 performed sufficiently well to recommend their use in the clinical setting of critically ill patients (area under the receiver operating characteristic curve ≤0.65). This study shows the poor performance of standard urinary indices at ICU admission for differentiating transient AKI from persistent AKI in unselected critically ill patients. Although changes over the first 24 hours and values at H24 performed slightly better than values at admission, their performance remained too low to be clinically useful. Together with other work [10], this study raises more fundamental questions about the value, meaning and pathophysiologic validity of the pre-renal AKI paradigm and suggests that AKI (like all other forms of organ injury) is a continuum of injury that cannot be neatly divided into functional (pre-renal or transient) or structural (acute tubular necrosis or persistent).

In the near future, biomarkers of renal dysfunction may identify additional patients with AKI and may identify the majority of patients at an earlier stage. Among biomarkers of AKI, the largest body of evidence for the detection of AKI is related to both urine and plasma neutrophil gelatinase associated lipocain (NGAL) [13-15]. Di Somma and coauthors [16] demonstrated, in a multicenter Italian prospective emergency department cohort study enrolling 665 patients admitted to hospital from the emergency department, that assessment of a patient’s initial blood NGAL improved the initial clinical diagnosis of AKI, predicting the in-hospital mortality. They found that blood NGAL assessment coupled with the emergency department physician’s clinical judgment may prove useful in deciding the appropriate strategies for patients at risk for the development of AKI.

In a provocative paper, Kashani and colleagues [17] found that the concentration of two novel markers - insulin-like growth factor-binding protein 7 and tissue inhibitor of metalloproteinases-2 - was increased in the urine of patients at high risk of AKI from a variety of etiologies. These authors then validated these markers in a separate second phase of their study and compared them with known markers of AKI such as NGAL and kidney injury marker-1. Not only did each marker perform better than other known markers, but their combination improved risk stratification when added to a complex multi-variable clinical model including age, serum creatinine, Acute Physiology and Chronic Health Evaluation III score, hypertension, nephrotoxic drugs, liver disease, sepsis, diabetes, and chronic kidney disease. After definitive clinical validation, the application of novel AKI biomarkers will permit appropriate triage of patients, more intensive monitoring, and perhaps early involvement of specialists in nephrology and critical care who can promptly evaluate these patients while they are still in the golden hours of this disease prior to irreversible damage to the kidneys. Similar to cardiac troponin, renal biomarkers are expected in the coming years to allow timely, bedside, sensitive and specific diagnosis of renal dysfunction, even in the emergency room. Currently, however, the ‘renal troponin’ has not been found and patient evaluation, including global examination of medical history, routinely available laboratory examinations and clinical signs, remain the mainstay of AKI diagnosis.

## Risk factors and hemodynamics of acute kidney injury

Kellum and Lameire [1] proposed the concept of risk factors and susceptibility. The main external stressors that may cause AKI include sepsis, shock, burns, trauma, cardiopulmonary bypass, nephrotoxic drugs, radiocontrast agents, and poisonous plants and animals. However, the chances of developing AKI after exposure to the same insult depend on a number of ‘susceptibility factors’ that vary widely from individual to individual. For susceptibility factors we may consider advanced age, dehydration or volume depletion, female gender, black race, chronic kidney disease, diabetes mellitus, cancer and anemia. Therefore, it is important to screen patients who have undergone an exposure and to continue monitoring them until the risk has subsided. To better interpret the pathophysiology of AKI and its interplay with several hemodynamic variables, a retrospective study including 137 septic ICU patients was performed [18]: AKI was defined as occurrence of a novel AKI case or an increase in the stage of previously diagnosed AKI during the first 5 days following ICU admission based on the AKIN criteria. Central venous pressure (CVP), cardiac output, mean arterial pressure (MAP), diastolic arterial pressure (DAP), central venous oxygen saturation (ScvO_2_) or mixed venous oxygen saturation were analyzed. One-half of patients in the study had new or persistent AKI. MAP, ScvO_2_ and cardiac output were not significantly different between AKI and non-AKI patients. The development or progression of AKI, regardless of the level of fluid balance and positive end-expiratory pressure, was strongly associated with the CVP level. This suggests participation of venous congestion in the physiopathology of AKI in severe sepsis and septic shock. Patients with AKI had lower DAP and higher CVP (*P* = 0.0003). Although the role of renal hypoperfusion (low cardiac output or hypovolemia) is believed to contribute to the development of sepsis-induced renal dysfunction, AKI appears to be only partially reversible after the optimization of systemic hemodynamics [18]. Fluid resuscitation and pressure optimization is a landmark treatment for septic patients in order to improve renal perfusion pressure. For some patients, the induced CVP elevation may overcome the DAP increase, reducing renal perfusion with harmful effects on renal function. This aspect is supported by the recently reported association between fluid overload and mortality in critically ill patients, especially in patients with AKI or septic shock [19]. The creation of a vicious circle with oliguria and fluid-loading may then aggravate AKI. Therefore, targeting a pre-defined CVP as a therapeutic target might not be suitable in septic patients. Legrand and colleagues suggested instead that hemodynamic targets are best achieved at low CVPs (that is, a CVP less than 8 to 12 mmHg) [18]. The Surviving Sepsis Campaign guidelines [20] mention that ‘in mechanically ventilated patients or those with known preexisting decreased ventricular compliance, a higher target CVP of 12–15 mmHg should be achieved to account for the impediment in filling’. The results of this study suggest, instead, that such targets might be too high from a ‘renal’ perspective. Therefore, a strategy of fluid restriction in these patients is an important option to be considered.

Poukkanen and coworkers [21] evaluated if a higher MAP maintained during the first 24 hours of ICU admission is associated with a lower risk of progression of AKI in patients with severe sepsis. More than 400 patients with severe sepsis were enrolled in this prospective observational study. The primary endpoint was progression of AKI within the first 5 days of ICU admission defined as new onset or worsening of AKI by the KDIGO criteria. AKI progressed in 153 patients (36.2%) and these patients had significantly lower time-adjusted MAP (74.4 mmHg (68.3 to 80.8)), than those without progression (78.6 mmHg (72.9 to 85.4)) (*P* <0.001). A cutoff value of 73 mmHg for time-adjusted MAP best predicted the progression of AKI. These authors also found that chronic kidney disease, higher lactate, higher dose of furosemide, use of dobutamine and time-adjusted MAP below 73 mmHg were independent predictors of progression of AKI. Interestingly, a more recent randomized controlled trial assigning septic shock patients to arms with MAP targets of 80 to 85 mmHg (high-target group) or 65 to 70 mmHg (low-target group) found renal outcome differences only in patients with chronic hypertension [22]. The main difference between the observational study of Poukkanen and colleagues and the prospective SEPSISPAM study is that, in the first, hypotensive patients were those with the highest vasopressor loads and, in the second, the high target group received the largest amount of vasoactive drugs. It can only be concluded that, possibly, the underlying septic syndrome severity rather than MAP or inotropic score is the most important determinant of renal function.

## Outcome

The impact of AKI on long-term clinical outcomes still remains controversial. Many of the studies evaluating the outcome of AKI patients come from the cardiac ICU where the model of AKI has already been well established. However, long-term outcomes in a larger setting of patients still remain to be fully evaluated. For this purpose, Hansen and coworkers [23] conducted a cohort study including 1,030 patients scheduled for acute or elective cardiac surgery in order to examine the 5-year risk of death, myocardial infarction, and stroke after elective cardiac surgery complicated by AKI. AKI was defined using the AKIN criteria. Patients where followed from the fifth post-operative day until myocardial infarction, stroke or death happened within 5 years. A total of 287 (27.9%) of 1,030 patients developed AKI. The 5-year risk of death was 26.5% (95% confidence interval (CI) 21.2 to 32.0) among patients with AKI and 12.1% (95% CI 10.0 to 14.7) among patients without AKI. The corresponding adjusted hazard ratio (HR) of death was 1.6 (95% CI 1.1 to 2.2). The 5-year risk of myocardial infarction was 5.0% (95% CI 2.9 to 8.1) among patients with AKI and 3.3% (95% CI 2.1 to 4.8) among patients without AKI. The 5-year risk of stroke was 5.0% (95% CI 2.8 to 7.9) among patients with AKI and 4.2% (95% CI 2.9 to 5.8) among patients without AKI. Adjusted HRs were 1.5 (95% CI 0.7 to 3.2) for myocardial infarction and 0.9 (95% CI 0.5 to 1.8) for stroke. AKI within 5 days after elective cardiac surgery was associated with increased 5-year mortality but not with increased risk of myocardial infarction or stroke.

In line with these results the work of Lopez-Delgado and coworkers [24] evaluated the impact of AKI on short- and long-term outcome (6.9 ± 4.3 years) after cardiac surgery. This group prospectively studied 2,940 consecutive cardio-surgical patients and AKI was defined according to the modified RIFLE criteria; 14% (n = 409) of the enrolled patients were diagnosed with AKI. One intra-operative (longer cardiopulmonary bypass time) and two post-operative (a longer need for vasoactive drugs and higher arterial lactate level 24 hours after admission) variables were identified as predictors of AKI. The worst outcomes, including in-hospital mortality, were associated with the worst RIFLE class. Kaplan-Meier analysis showed survival of 74.9% in the RIFLE risk group, 42.9% in the RIFLE injury group and 22.3% in the RIFLE failure group (*P* <0.001). Classification at RIFLE injury (HR 2.347, 95% CI 1.122 to 4.907, *P* = 0.023) and RIFLE failure (HR 3.093, 95% CI 1.460 to 6.550, *P* = 0.003) were independent predictors for long-term patient mortality. These authors concluded that AKI development after cardiac surgery is associated with post-operative variables, which ultimately could lead to a worse RIFLE class. Staging at the RIFLE injury and RIFLE failure classes is associated with higher short- and long-term mortality in this population. It remains to be ascertained if these data on AKI patient outcomes may be applicable to non-cardiac surgery critically ill patients.

## Renal replacement therapy

In critically ill patients with AKI, urea and creatinine are not well performing indicators of renal function given the lack of steady state in terms of production and the influence of catabolism, volume status and production rates, particularly in sepsis. Consequently, physicians treating critically ill patients put an increasing emphasis on fluid overload, oliguria, impaired oxygenation and acidosis as triggers for initiation of renal replacement therapy (RRT) with a general trend to initiate RRT earlier in sicker patients. Thakar and colleagues [25] performed an international survey predominantly among North American nephrologists consulting in the ICU. They evaluated practice patterns for the initiation of RRT using three different scenarios representing patients with increased severity of disease. The majority of the 172 respondents (70% USA) expressed a reticence in commencing RRT early given the lack of evidence, preferring to base their decision on absolute levels of creatinine or blood urea nitrogen (BUN) (>442 μmol/l (>5 mg/dl), >35.6 mmol/l (>100 mg/dl), respectively) rather than any relative rise. Despite this, 94% of physicians reported that they would be likely to start dialysis early in patients with the highest disease burden with early RRT described as a lower BUN on commencing treatment. Thus, the proportion of physicians starting dialysis at a BUN <75 mg/dl tripled in the more severe case. Finally, given a selection of five parameters (BUN, creatinine, urine output, oxygenation and potassium) for starting RRT, the latter two were given the highest priority, with oxygen saturation appearing as the most frequent trigger in severe cases. These results also differ from a recently published survey among 275, mainly European, intensivists [26]. Whereas the median thresholds with regard to sCr (300 μmol/l) or urea (40 mmol/l) were similar, there was a higher priority with hyperkalemia, metabolic acidosis and volume overload. In addition, they demonstrated a trend towards early initiation of RRT, with the majority favoring initiation when a diagnosis of AKI was made based on AKIN/RIFLE criteria, particularly with regard to oliguria [27]. As usual, it must be remarked that data from surveys have a high risk of self-selection of participants; however, these results should not be overlooked as they represent the view of a large panel of experts.

Data from observational studies have suggested that early RRT in critically ill patients with AKI may have a beneficial impact on survival [28,29]. However, in addition to the lack of large randomized clinical trials assessing early RRT indication, there is a broad variation in the criteria used to classify early or late RRT.

For this purpose Leite and coworkers [30], using the AKIN classification, compared RRT initiation in critically ill patients, defining early or late RRT in reference to timing after stage 3 AKIN. Patients beginning RRT within 24 hours after AKI stage 3 were considered early starters. AKIN criteria were evaluated by both urine output and sCr. Patients with acute-on-chronic kidney disease were excluded from this study. A total of 358 critically ill patients were managed with RRT but only 150 patients with AKI at stage 3 were analyzed: mortality was lower in the early RRT group (51.5 versus 77.9%, *P* = 0.001). After achieving balance between the groups using a propensity score, there was a significant 30.5% (95% CI 14.4 to 45.2%, *P* = 0.002) relative decrease of mortality in the early RRT group. Moreover, patients in the early RRT group had lower duration of mechanical ventilation and time on RRT and a trend to lower ICU length of stay. Although showing promising results in terms of performing early RRT in critically ill patients, this study evaluated only AKIN stage 3, so the influence of RRT on earlier AKI stages was not fully evaluated. Another limitation is the lack of data on fluid balance, making it difficult to make conclusions about reduced mechanical ventilation time in early RRT patients.

Another important aspect investigated in *Critical Care* is the choice of the most appropriate modality of renal replacement therapy for AKI management in critically ill patients [31]. In current practice many different approaches are used in the ICU. One basic and important issue is the frequency of RRT delivery: in terms of hard outcomes (namely mortality and length of hospital stay) it has currently not been clarified if dialysis should be delivered in the ICU in a continuous or intermittent fashion. The single-center prospective randomized clinical trial Comparing Continuous Versus Intermittent Hemodialysis in ICU Patients (CONVINT) included 252 critically ill patients with dialysis-dependent AKI [32]. Patients were randomized to receive either daily intermittent hemodialysis (IHD) or continuous veno-venous hemofiltration (CVVH). The primary outcome measure was survival at 14 days after the end of RRT. Secondary outcome measures included 30-day, ICU, and intra-hospital mortality, as well as course of disease severity/biomarkers and need for organ-support therapy. Survival rates at 14 days after RRT were 39.5% (IHD) versus 43.9% (CVVH) (odds ratio 0.84, 95% CI 0.49 to 1.41, *P* = 0.50). No differences were observed for days on RRT, vasopressor days, days on ventilator, or ICU/intra-hospital length of stay. After the results of this study, intermittent and continuous RRT may be considered equivalent approaches for critically ill patients with dialysis-dependent acute renal failure. As the accompanying editorial also commented [31], both modalities should probably be available in the ICU and the best option considered each time a patient’s clinical condition is re-assessed: it is possible that more stable patients may gain advantage from short sessions of IHD, whereas those on the critical side of ICU management (requiring administration of frequent volume boluses or vasopressor drugs) may still find benefit from continuous RRT (CRRT).

## Extracorporeal oxygenation/decapneization and the kidney

The impact of AKI during extracorporeal membrane oxygenation (ECMO) and the use of CRRT to remove CO_2_ during the management of moderate forms of acute respiratory distress syndrome (ARDS) are two topics evaluated by expert investigators in the field of critical care nephrology in 2013.

The impact of AKI in a selected population of children (neonates) has been well discussed by Zwiers and coworkers [33] from Rotterdam, Sophia Children’s Hospital. They reviewed 14 years of prospectively collected clinical data, including age, diagnosis, ECMO course and sCr of all ECMO-treated neonates within their institution. ECMO is an advanced extracorporeal technique providing life support (cardiac and/or respiratory) to patients with acute reversible respiratory or cardiovascular failure who are not responding to conventional intensive care. Zwiers and colleagues used the pediatric RIFLE categorization to better identify patients suffering from AKI. This cohort study included 242 critically ill neonates receiving ECMO support, 179 (74%) of whom survived. In total, 153 (64%) patients had evidence of AKI, with 72 (30%) qualifying as RIFLE Risk, 55 (23%) as RIFLE Injury, and 26 (11%) as RIFLE Failure. At the end of the study period, only 71 (46%) of the 153 AKI patients improved by at least one pediatric RIFLE category. Survival until ICU discharge was significantly lower for patients in the failure category (35%) compared with the non-AKI (78%), risk (82%), and injury (76%) categories (*P* <0.001), whereas no significant differences were found between the three latter RIFLE categories. This study did have some limitations, such as: a) the sCr level is a delayed measure of decreased kidney function after AKI and is not very sensitive; b) the ECMO circuit in neonates doubles the circulating volume, thereby diluting sCr levels - thus, the true incidence of AKI during the first days of ECMO treatment is hard to establish; c) the urine output for grading AKI was not used in this study and the results acquired could not, therefore, be accurate according to pediatric RIFLE classification. Since AKI during childhood may predispose to chronic kidney disease in adulthood, we believe that long-term monitoring of kidney function after neonatal ECMO should be mandatory. As provocatively addressed in the interesting accompanying editorial, AKI in the course of multiple organ dysfunction syndrome (MODS) remains a ‘circular reference’ [34]: AKI in patients requiring ECMO therapy is triggered by initial systemic insults, already present at the time of ECMO initiation, and aggravated by reperfusion injury and ECMO circuit-induced inflammation. The close interplay between the cause and severity of the disease, supportive therapies, and host response may significantly influence the development of AKI and MODS. Identifying as well as determining the relative importance of extrinsic factors aggravating MODS/AKI during ECMO therapy is central: circuit biocompatibility, monitoring organ ‘reperfusion’ as well as function (through the implementation of novel organ-specific or injury-specific biomarkers), and deleterious therapeutic interventions, such as antimicrobial chemotherapy administered in AKI patients without a thorough drug level control [34].

The second important technological aspect covered in 2013 is the use of CRRT systems coupled with a CO_2_ removal device for the management of moderate forms of ARDS. Mechanical ventilation using high tidal volumes and high airway pressures has been shown to be deleterious for patient outcomes; thus, protective ventilation strategies, including lower tidal volumes, have been implemented in clinical practice [35]. Although this strategy may lead to respiratory acidosis, a careful and gentle ventilation strategy with permissive hypercapnia and concomitant slight acidosis is presently widely accepted. The exact threshold to which respiratory acidosis should be tolerated is presently a matter of debate: in the most severe cases, ECMO and pumpless extracorporeal lung assist devices are increasingly used to support lung-protective ventilation strategies and to improve CO_2_ removal. Forster and coworkers [35] tried to assess the effectiveness of a hollow-fiber gas exchanger integrated into a conventional renal-replacement circuit on CO_2_ removal, acidosis and hemodynamics. In 10 ventilated critically ill patients with ARDS and AKI undergoing renal and respiratory support, the effects of low-flow CO_2_ removal on respiratory acidosis compensation were tested: CO_2_ elimination in the low-flow circuit was safe and was well tolerated by all patients. After 4 hours of treatment, a mean reduction in arterial CO_2_ of 17.3 mmHg (−28.1%) was observed, in line with an increase in pH. In hemodynamically unstable patients, low-flow CO_2_ elimination was paralleled by hemodynamic improvement, with an average reduction of vasopressors of 65% in five of six catecholamine-dependent patients during the first 24 hours. Integration of a hollow-fiber gas exchanger could thus potentially be an additional tool in the armamentarium of treatment modalities in patients with multiorgan failure. Because no further catheters are needed, besides those for renal replacement, the implementation of a hollow-fiber gas exchanger in a renal circuit could be an attractive and minimally invasive therapeutic tool; it remains to be specified whether the application of these devices should be limited to patients with mild to moderate ARDS, suffering from prevalently CO_2_ accumulation and no severe oxygenation defects.

## Conclusion

Interesting new ideas were evaluated and addressed in 2013 in the field of critical care nephrology: new classification criteria (KDIGO), new promising biomarkers (insulin-like growth factor-binding protein 7 and tissue inhibitor of metalloproteinases-2), new concepts on timing of RRT and the importance of fluid overload. Furthermore, current research in this field is increasingly focusing on the (unfortunately poor) long-term follow-up of critically ill patients who have suffered from severe AKI. Finally the continuous implementation in clinical practice of multiple organ support therapy (that is, ECMO or decarboxylation devices adapted to a CRRT platform) further highlights the great development of extracorporeal treatments for the care of more complex and challenging patients.

## Note: 

This article is part of a collection of Year in review articles in *Critical Care*. Other articles in this series can be found at http://ccforum.com/series/Yearinreview2013.

## Abbreviations

AKI: Acute kidney injury
AKIN: Acute Kidney Injury Network
ARDS: Acute respiratory distress syndrome
BUN: Blood urea nitrogen
CI: Confidence interval
CRRT: Continuous renal replacement therapy
CVP: Central venous pressure
CVVH: Continuous veno-venous hemofiltration
DAP: Diastolic arterial pressure
ECMO: Extracorporeal membrane oxygenation
FeUrea: Fractional excretion of urea
HR: Hazard ratio
IHD: Intermittent hemodialysis
KDIGO: Kidney disease: improving global outcome
MAP: Mean arterial pressure
MODS: Multiple organ dysfunction syndrome
NGAL: Neutrophil gelatinase associated lipocalin
RIFLE: Risk, injury, failure, loss of function and end stage kidney disease
RRT: Renal replacement therapy
sCr: Serum creatinine
ScvO_2_: Central venous oxygen saturation
